# An illness-focused interactive booklet to optimise management and medication for childhood fever and infections in out-of-hours primary care: study protocol for a cluster randomised trial

**DOI:** 10.1186/s13063-016-1667-8

**Published:** 2016-11-17

**Authors:** Eefje G. P. M. de Bont, Geert-Jan Dinant, Gijs Elshout, Gijs van Well, Nick A. Francis, Bjorn Winkens, Jochen W. L. Cals

**Affiliations:** 1 Department of Family Medicine, CAPHRI School for Public Health and Primary Care, Maastricht University, P.O. Box 616, 6200 MD Maastricht, The Netherlands; 2 Department of General Practice, Erasmus MC, University Medical Centre Rotterdam, P.O. Box 2040, 3000 CA Rotterdam, The Netherlands; 3 Department of Paediatrics, Maastricht University Medical Centre (MUMC+), P.O. Box 5800, 6212 AZ Maastricht, The Netherlands; 4 Division of Population Medicine, School of Medicine, Cardiff University, CF14 4YS Cardiff, UK; 5 Department of Methodology and Statistics, CAPHRI School for Public Health and Primary Care, Maastricht University, P.O. Box 616, 6200 MD Maastricht, The Netherlands

**Keywords:** Antibiotics, Child, Primary care, Booklet, General practitioner, Out-of-hours

## Abstract

**Background:**

Fever is the most common reason for a child to be taken to a general practitioner (GP), especially during out-of-hours care. It is mostly caused by self-limiting infections. However, antibiotic prescription rates remain high, especially during out-of-hours care. Anxiety and lack of knowledge among parents, and perceived pressure to prescribe antibiotics amongst GPs, are important determinants of excessive antibiotic prescriptions. An illness-focused interactive booklet has the potential to improve this by providing parents with information about fever self-management strategies. The aim of this study is to develop and determine the effectiveness of an interactive booklet on management of children presenting with fever at Dutch GP out-of-hours cooperatives.

**Methods/design:**

We are conducting a cluster randomised controlled trial (RCT) with 20 GP out-of-hours cooperatives randomised to 1 of 2 arms: GP access to the illness-focused interactive booklet or care as usual. GPs working at intervention sites will have access to the booklet, which was developed in a multistage process. It consists of a traffic light system for parents on how to respond to fever-related symptoms, as well as information on natural course of infections, benefits and harms of (antibiotic) medications, self-management strategies and ‘safety net’ instructions. Children < 12 years of age with parent-reported or physician-measured fever are eligible for inclusion. The primary outcome is antibiotic prescribing during the initial consultation. Secondary outcomes are (intention to) (re)consult, antibiotic prescriptions during re-consultations, referrals, parental satisfaction and reassurance. In 6 months, 20,000 children will be recruited to find a difference in antibiotic prescribing rates of 25% in the control group and 19% in the intervention group. Statistical analysis will be performed using descriptive statistics and by fitting two-level (GP out-of-hours cooperative and patient) random intercept logistic regression models.

**Discussion:**

This will be the first and largest cluster RCT evaluating the effectiveness of an illness-focused interactive booklet during GP out-of-hours consultations with febrile children receiving antibiotic prescriptions. It is hypothesised that use of the booklet will result in a reduced number of antibiotic prescriptions, improved parental satisfaction and reduced intention to re-consult.

**Trial registration:**

ClinicalTrials.gov identifier: NCT02594553. Registered on 26 Oct 2015​, last updated 15 Sept 2016.

**Electronic supplementary material:**

The online version of this article (doi:10.1186/s13063-016-1667-8) contains supplementary material, which is available to authorized users.

## Background

Fever is the most common reason for a child to be taken to a general practitioner (GP). Childhood infections constitute 60% of the annual general practice consultation rates for children younger than 1 year old and approximately 30% for children up to 15 years of age [1]. These rates are even higher during out-of-hours care because fever typically rises during the day [1–3].

In most cases, fever is caused by a benign (viral) infection, and general recommendations given by the GP are sufficient. However, one in three to four children who visit a GP out-of-hours centre because of a fever receive an antibiotic prescription. Most often, this is unnecessary and not recommended in guidelines [4, 5]. Additionally, these prescription rates are nearly twice as high as prescription rates during routine office hours [6].

Previous studies showed that antibiotic prescribing is strongly influenced by patients’ expectations and that GPs experience pressure from patients to prescribe antibiotics [7]. Parents who visit a GP are often concerned about harmful consequences of fever and serious infections, especially when presenting to a GP on call who is not their personal GP. In many cases, these concerns are the result of these parents’ lack of experience and knowledge about fever [2]. Their worries are increased by a rising temperature but also by conflicting information on how to manage fever from different health care providers, websites or people in their surroundings [8]. Parents search for reassurance, especially when fever is accompanied by other symptoms.

Although GPs sometimes feel pressured to prescribe antibiotics, most parents of a febrile child in fact do not expect antibiotics. They are, however, in search of reassurance and consistent, reliable information about fever, specific symptoms and self-management strategies [9]. Nevertheless, conveying evidence-based information to patients on the cause of symptoms, natural course of the symptoms, and the expected benefits and harms of treatment is challenging for GPs, especially in time-pressured consultations in the evening and night [10]. GPs perceive that children with a fever account for a high workload during out-of-hours care [11]. This can lead to frustration and a diagnostic challenge due to the low incidence of serious conditions and a lacking long-term relationship during out-of-hours care. These factors play an important role in GPs’ decisions when they prescribe antibiotics to children during out-of-hours care because only few children do have a serious infection such as pneumonia, meningitis or complicated urinary tract infection. Concern about missing these serious infections helps drive fear, consulting and prescribing behaviour. However, empowering parents and teaching them alarm symptoms minimises the risk of missing serious infections and helps to not routinely prescribing antibiotics [11].

Illness-focused interventions recognise the importance of non-medical influences on the decision to consult or to prescribe antibiotics. Exploring the illness experience of parents of children with fever and infections may have potential because it specifically addresses the concerns and questions that parents have when their child is sick. Moreover, it may offer the GP a way to convey consistent written information, enhancing their self-management and providing them with ‘safety net’ advice when they return home with clear instructions in what case to return or seek contact again [12]. An illness-focused GP-parent information exchange tool consisting of an interactive booklet has the potential to provide parents with information about symptoms and fever management as well as consistent information during GP consultations [13, 14]. A strong safety net advice provided in a booklet can hypothetically also provide a disease-focused solution to GPs by providing them with a way to reduce diagnostic uncertainty with these children, thereby also reducing the number of ‘better safe than sorry’ antibiotic prescriptions [15].

In summary, anxiety and lack of knowledge among parents, as well as perceived pressure to prescribe antibiotics during time-pressured and diagnostically challenging consultations among GPs, are important determinants of excessive antibiotic prescriptions for febrile children and of inconsistencies in providing care to this vulnerable group of patients. The aim of the Childhood Infections Limburg (CHILI) study is therefore to develop and determine the effectiveness of an illness-focused interactive fever booklet for parents on the management (antibiotic prescriptions, [re-]consultations and intention to re-consult, referral rates, parental satisfaction and self-reported adverse events) of children presenting with fever at Dutch GP out-of-hours cooperatives, as well as on relevant parental outcomes (satisfaction and reassurance). It is hypothesised that the use of an interactive booklet during consultations for febrile children at GP out-of-hours centres will result in a reduced number of antibiotic prescriptions, improved parental satisfaction and reduced intention to re-consult.

## Methods/design

We will conduct a cluster randomised controlled trial (RCT) with randomisation on the level of GP out-of-hours cooperative. Recruited GP out-of-hours cooperatives will be randomised to one of two arms: GP access to the illness-focused interactive booklet or care as usual.

### Objectives

In this study, we will investigate the following research question: What is the effect of the pragmatic use of an interactive booklet in childhood fever related consultations for children <12 years, during GP out-of-hours care consultations on the primary and secondary outcome measures (outlined below)?

#### Primary outcome measure

1. Antibiotic prescribing rate during the initial consultation

#### Secondary outcome measures based on the complete sample

2. Re-consultation rate at the GP out-of-hours cooperative for the same illness episode within 2 weeks of the initial consultation
3. Antibiotic prescribing rate during re-consultations at the GP out-of-hours cooperative within 2 weeks of the initial consultation
4. Re-consultations for fever and fever-related conditions at the GP cooperative during out-of-hours care during the 6-month study period
5. Referral to secondary care during the initial consultation and for the same illness episode within 2 weeks of the initial consultation

#### Secondary outcome measures based on telephone survey

6. Parent-reported re-consultation rate at their own GP during routine daytime hours for the same illness episode within 2 weeks of the initial consultation
7. Parent-reported antibiotic prescribing during re-consultations at their own GP during routine daytime hours for the same illness episode within 2 weeks of the initial consultation
8. Parent-reported hospital admission for that illness episode within 2 weeks of the initial consultation
9. Parent-reported satisfaction with care and parent-reported satisfaction with providing written information materials (including the interactive booklet)
10. Parent-reported intention to re-consult the out-of-hours GP centre for a future similar illness episode

### Development of the intervention

**Fig. 1:**
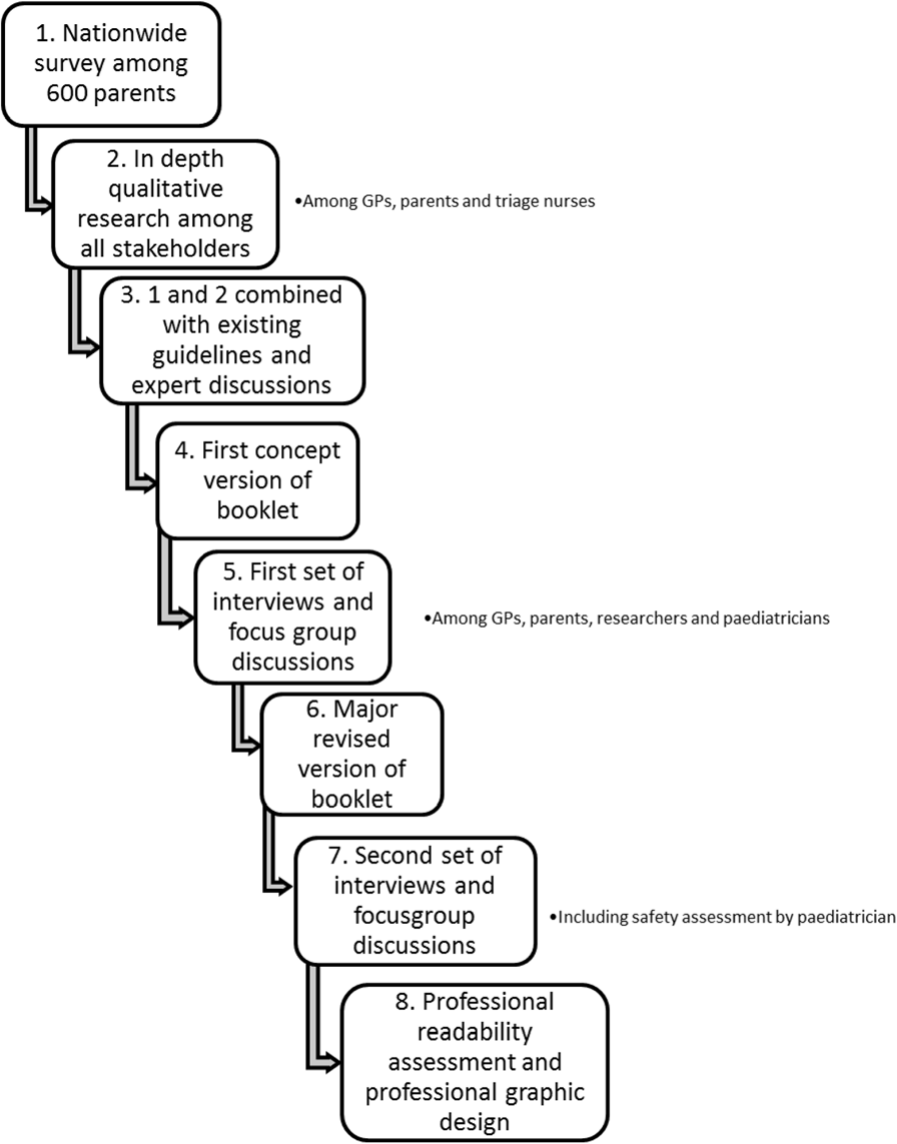
Overview of the development process of the interactive booklet. *GP* General practitioner

The illness-focused interactive booklet was developed in a multistage process (see Fig. 1). This process was based partially on the development of a previous booklet for upper respiratory tract infections in children that was proven to be effective [16]. First, a nationwide survey among parents of young children was conducted to obtain insight into parental knowledge, attitudes and practices regarding fever management [2]. Second, a more in-depth exploration of determinants and influencing factors of GP out-of-hours consultations was performed in focus group sessions and semi-structured interviews with parents, GPs and triage nurses working or consulting during out-of-hours GP care [9, 11]. Through this body of research, we identified a number of themes focused on ‘What do parents want when their child has a fever?’ and ‘What do GPs need to provide regarding evidence-based information during childhood fever consultations aimed at the illness experience of parents?’ We developed an illness-focused interactive booklet based on these themes, helped by existing guidelines and expert discussions. The booklet contains the following sections:

- A traffic light system for fever in general with advice on when to consult (red) and information on self-management strategies (green, orange) for childhood fever in general, as well as specific traffic lights for upper respiratory tract infections (cough, cold and sore throat), acute otitis media (earache) and gastrointestinal symptoms (abdominal pain, vomiting and diarrhoea), helping parents to know when to (re)consult and providing them with self-management strategies as well as a safety net
- Information on the benefits and harms of antibiotic treatment (helping parents to make a balanced choice between necessary and unnecessary/undesired uses of antibiotics)
- An overview of natural duration of common infections in children with a figure displaying the average duration as well as the number of days when 90% of children are free of symptoms (helping parents to set realistic expectations on how long their child’s illness may last)
- A table with weight-banded paracetamol dosage schemes (helping parents to provide their child with a safe yet effective dose of analgesics if these are required)
- Advice and information on febrile convulsions and skin rash (helping parents to recognise alarm symptoms and differentiate these from other benign and common symptoms)
- Safety net advice for fever in general as well as safety net advise for the different common infections (helping GPs to create a safety net and helping parents to act upon alarm symptoms so that children who do develop a serious infection are recognised without any delay and complications)

Semi-structured interviews were held to discuss pilot version of the booklet with GPs, paediatricians and parents. The booklet was then revised in accordance with the feedback that was agreed on by the research group. Subsequently, the readability of the booklet was assessed and, when necessary, adapted by a professional language expert, including an assessment specifically focused on readability for lay persons. After this, another round of semi-structured interviews was held. Finally, the layout of the booklet was professionally adapted.

The booklet incorporates existing information about fever, alarm symptoms, use of antipyretics and antibiotics, and specific infectious diseases that frequently occur in childhood in combination with fever, such as upper respiratory tract infections, otitis media, urinary tract infections and gastro-enteritis [17]. The content of this information is similar to the information which is already provided by GPs during care as usual. The main difference with these existing sources of information is the use of a traffic light system where symptoms and the advice belonging to those symptoms are incorporated in the categories green, orange and red, from most harmless to most urgent, respectively. Use of such a traffic light system can also be found in the international National Institute for Health and Care Excellence guideline, where the traffic light is disease-focused and aimed at health care professionals instead of parents [1]. The major difference is that this booklet is illness-focused, meaning it is specifically aimed at parents and their unique illness experience and decision to consult a GP. Making this information available in the consulting room may facilitate communication about caring for a febrile child and address misconceptions GPs still hold about parents and patients expecting antibiotics. The interactive part of the booklet therefore implies that it is designed to facilitate the exploration of the illness experience of parents of children with fever and common infections to help GPs to specifically address these concerns and questions that parents have when their child is sick and preventing ‘better safe than sorry antibiotic prescriptions’ in these consultations during out-of-hours care.

Our previous qualitative work among parents having visited out-of-hours care revealed that most parents are in search of consistent, reliable information about fever and specific symptoms, which they often do not find on the Internet. Most parents did not receive written information from their GP during the consultation, but most suggested that information about alarm symptoms and self-management strategies would be helpful and that it would be important that this information come from one comprehensible and reliable information source without inconsistencies. Hence, another major difference with current available information sources is the fact that all the information is incorporated into one booklet which can be physically handed to and discussed with parents.

### Setting

Since 2000, GP out-of-hours care in The Netherlands has been provided by approximately 120–130 large-scale GP cooperatives, varying from 50 to 200 GPs [18]. These cooperatives cover primary care by rotating shifts of GPs during evenings, nights and weekends. This means that in almost every consultation, GPs and parents or patients have not met in previous clinical encounters. *Out-of-hours care* is defined as primary care provided beyond office hours every day between 5 p.m. and 8 a.m. and the entire weekend [18]. GP out-of-hours centres are essentially intended for urgent help requests that cannot wait until the next day [19]. There will be 20 large, rural and urban GP out-of-hours centres participating in this study, spread across The Netherlands.

### Randomisation

**Fig. 2:**
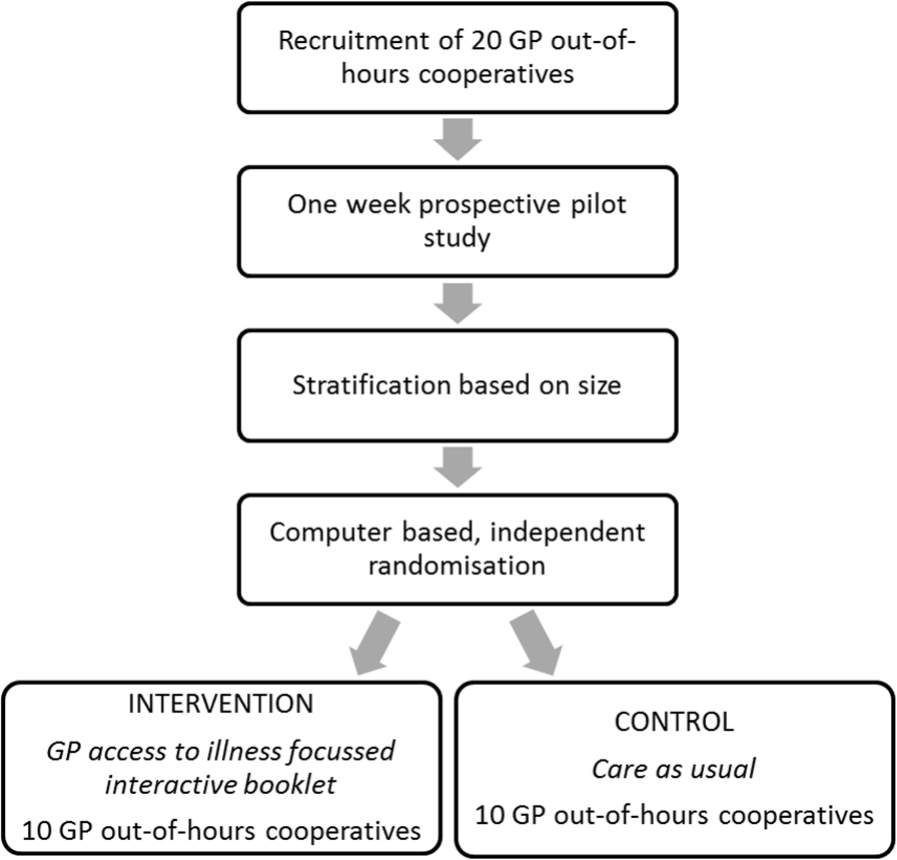
Graphical overview of randomisation and inclusion of the Childhood Infections Limburg (CHILI) project. *GP* General practitioner

A cluster RCT design was chosen to reduce the risk of contamination. On the basis of benchmark data provided by the national organisation of out-of-hours care (InEen), the participating GP out-of-hours cooperatives will be stratified by size (10 small vs. 10 large cooperatives, with a cut-off point of fewer or more than 20,500 consultations/year) to ensure equal distribution of size between the intervention and control groups. Should the stratification not result in two equal groups of ten, the cut-off point will be reconsidered. An independent researcher who is not involved in the project will perform computer-based randomisation. Random permuted blocks of two will be generated. This will create ten groups of two GP out-of-hours cooperatives and ensure equal distribution of the intervention and control situations. The randomisation process is graphically presented in Fig. 2. The randomisation outcome will be kept securely, and allocation for each cooperative will be provided only after the cooperative has agreed to participate and the stratification variables are provided to the independent researcher.

### Sample size

To inform the required sample size, we performed a retrospective cohort study to determine the number of children visiting a GP out-of-hours cooperative [4]. We identified 17,170 contacts for children younger than 12 years of age. Of these, 5343 (31.1%) were fever-related, and 70% of these fever-related contacts resulted in a face-to-face consultation based on data of all contacts of 1 cooperative providing care to approximately 270,000 inhabitants. This led to a total of 3738 consultations during 1 year and an average of 15 consultations per day for children with fever and fever-related conditions. The average antibiotic prescription rate we found during this cohort study was 25%, which we set as our baseline prescription rate. Additionally, in those out-of-hours centres which consented to participate in the trial, we performed a pilot study of 1 week to further investigate consultation rates, with the main consideration that GP out-of-hours centres do vary in size. During this pilot study, we found an average of six fever-related consultations for children per day per out-of-hours centre. On the basis of the pilot study and the retrospective cohort study, we assumed that 1000 children per cooperative could be included in 6 months, including the peak infection winter months.

The primary outcome is the antibiotic prescribing rate during the initial consultation (dichotomous). The required number of clusters and participants was based on the following assumptions: (1) intra-cluster correlation coefficient (ICC) of 0.01, based on a study that describes the distribution of intra-class correlation coefficients with reference to research in primary care [20]; (2) alpha of 0.05, power of 0.80; (3) proportion of antibiotic prescriptions in the control group of 25% and a proportion of 19% in the intervention group (6% minimal clinical relevant difference), based on the fact that we would thereby reduce the number of children receiving an antibiotic prescription from one in four to one in five to six; and (4) 10% loss to follow-up and 10% efficiency loss based on unequal cluster sizes [21]. We estimated we would need to include 1000 children per cluster (GP out-of-hours cooperative) within 6 months, resulting in a need for 20 clusters to acquire the same power as an individual RCT (with an effective sample size of 737 patients in both the intervention and control groups; 1474 in total). Hence, the total recruitment target for this cluster RCT is 20.000 children recruited at 20 GP out-of-hours centres (10 control, 10 intervention). The chosen reduction in antibiotic prescribing of 6% is arbitrary, and one could consider that any reduction in antibiotic prescribing that results from a low-cost, easy-to-implement intervention is clinically relevant in an era of rising antibiotic resistance.

### Recruitment

We will recruit 20 GP out-of-hours cooperatives that are going to participate and cluster randomise them either to GP access to the illness-focused interactive booklet or to care as usual (see Table 1). All GPs working at the participating GP out-of-hours centres that are in the intervention group (interactive booklet) will be introduced to the study content. GPs are subsequently instructed and trained by means of written instructions on how to use the booklet during consultations.

**Table 1:**
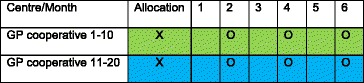
Overview implementation booklet using a cluster randomised controlled trial according to Standard Protocol Items: Recommendations for Interventional Trials (SPIRIT) guidelines

*Green* represents the control groups and *blue* the intervention groups. Baseline measurements will take place before implementation of the intervention. Measurement of the primary outcome and secondary outcomes based on the complete sample will go on automatically during the complete study period. The O represents a period of 2 weeks during which data on secondary outcomes based on the telephone survey will be collected

All cooperatives that will be recruited have to be working with the software system that will be used for data collection (Call Manager; Labelsoft Clinical IT B.V., CompuGroup Medical AG, Phoenix, AZ, USA). We will further specifically recruit cooperatives based on (1) their geographical location in The Netherlands to ensure widespread recruitment across the country and (2) the socio-economic status of the community in which they are providing care, to ensure that the sample will be representative of the rest of the country.

The booklet will be used during consultations with febrile children at the GP out-of-hours cooperative. The child’s symptoms will determine which information and advice parents receive from the GP. Inclusion criteria are age between 3 months and 12 years *and* the GP deciding this is a fever-related consult. The child’s temperature has to be measured by parents in advance of the consultation or by GPs during consultations. We specifically choose a subjective term for fever and not a temperature cut-off point, because parents’ considering their child to have a fever is, in our opinion, just as important as an actual clinical fever in light of the illness-focused intervention in this study.

The primary outcome data will be collected in a coded, automatic manner and will be supplied by an independent party that is responsible for the electronic patient file software (Labelsoft Clinical IT B.V.). Because providing written information about a disease can be considered a variation of care as usual, and because we will not be able to trace the data back to individual patients, the ethics committee waived the requirement of obtaining written informed consent during the consultation. Registration of the primary outcome (antibiotic prescriptions during the initial consultation) and secondary outcomes based on the complete sample ([re-]consultations during out-of-hours care, antibiotic prescriptions during re-consultations at the GP out-of-hours cooperative, and referral to secondary care) will be based on the electronic database. Parents and GPs are informed about the study through posters at the out-of-hours centre. We will also collect data on secondary outcomes using a telephone survey (intention to re-consult, parental satisfaction with care and the booklet, antibiotic prescriptions during re-consultations at their children’s personal GP, and self-reported adverse events) at three moments during a period of 2 weeks during months 2, 4 and 6. During these 2-week periods, parents of febrile children participating in the main study will receive a letter from the triage nurse in the waiting room explaining the study content. Parents are asked to provide informed consent to participate in a telephone survey after 2 weeks. Participation in the telephone survey will be completely voluntary, meaning parents in the intervention group will receive the booklet during their consultation, regardless of whether they consent to participate in the telephone survey.

### Outcome measurement

#### Primary outcome measure

1. Antibiotic prescribing rate during the initial consultation (baseline/index consultation, dichotomous scale; number of participants with an antibiotic prescription)

#### Secondary outcome measures based on the complete sample

2. Re-consultation rate at the GP out-of-hours cooperative for the same illness episode within 2 weeks of the initial consultation (within 2 weeks of initial consultation, number of re-consultations)
3. (Antibiotic) prescribing rate within 2 weeks of the initial consultation (hence including antibiotic prescriptions during re-consultations) at the GP out-of-hours cooperative (within 2 weeks of initial consultation, dichotomous scale; number of participants with an [antibiotic] prescription)
4. (Re-)consultations for fever and fever-related conditions at the GP cooperative during out-of-hours care during the 6-month study period (during complete study period of 6 months, number of consultations and re-consultations)
5. Referral to secondary care during the initial consultation and for the same illness episode within 2 weeks of the initial consultation (during index consultation and re-consultations within 2 weeks, number of referrals)

#### Secondary outcome measures based on the telephone survey

6. Parent-reported re-consultation rate at their own GP during routine daytime hours for the same illness episode within 2 weeks of the initial consultation (during telephone survey within 2 weeks of initial consultation, number of self-reported re-consultations)
7. Parent-reported antibiotic prescribing during re-consultations at their own GP during routine daytime hours for the same illness episode within 2 weeks of the initial consultation (during telephone survey within 2 weeks of initial consultation, dichotomous scale; number of self-reported [antibiotic] prescriptions)
8. Parent-reported hospital admission for that illness episode within 2 weeks of the initial consultation (during telephone survey within 2 weeks of initial consultation, dichotomous scale; number of self-reported hospital admissions)
9. Parent-reported satisfaction with care and with provision of written informational materials (during telephone survey within 2 weeks of initial consultation, dichotomous scale and visual analogue scale [VAS] score 1–10 on reassurance and satisfaction with care)
10. Parent-reported intention to re-consult the out-of-hours GP centre for a future, similar illness episode (during telephone survey within 2 weeks of initial consultation, dichotomous scale; number of parents with intention to re-consult for a future, similar illness)

### Data collection

During the complete study period from November 2015 to May 2016, we will collect anonymised data on baseline characteristics, antibiotic prescriptions, consultation rates and direct referrals to secondary care for febrile children from GP out-of-hours centre databases (Table 1). This is the complete study sample. Every time the GP processes patient information for a consultation of a child younger than 12 years of age, a pop-up screen will be displayed. GPs then have to answer the question, ‘Did this child have a fever (at home or at the GP cooperative)?’ (yes/no). The International Classification of Primary Care coding system will be used to map reasons for consultation. GPs in the intervention group will receive an additional pop-up after completing the consultation, to check whether or not they handed out the booklet. Because the primary outcome data will be collected in a coded, automatic manner and will be supplied by an independent party that is responsible for the electronic patient file software, there will be no data monitoring committee.

Data on secondary outcomes will be collected for a subsample using telephone surveys during three 2-week periods (Table 1). This will include parents of children also included in the main study. A triage nurse will provide parents with information about the study during their visits in these weeks. If parents give their consent, they will be asked to participate in a telephone survey 2 weeks after the initial consultation. Telephone surveys will be used to question parents about intention to re-consult in the same fever episode and in the future (yes/no), if they received and used antibiotics at re-consultation (yes/no), parental satisfaction (VAS scale), parental reassurance (reassured/not reassured and VAS scale), self-reported complications such as hospital admissions, consultations with their own GP before and after the out-of-hours consultation, and their opinion about the booklet (VAS scale, intention to use again, most important section). Measurements will take place during months 2, 4 and 6. The telephone survey data on secondary outcomes will be entered into a Microsoft Access database (Microsoft, Redmond, WA, USA) by two researchers independently.

### Analysis

First, the data will be processed with IBM SPSS Statistics for Windows software (IBM, Armonk, NY, USA), using mainly descriptive statistics to summarise the data. Second, statistical analysis will be based on the intention-to-treat principle by fitting two-level (GP out-of-hours cooperative and patient) random intercept logistic regression models using MLwiN software. Fixed parameters will be group (intervention vs. control) and size (small vs. large cooperative). The clustering in the data will be accounted for by a random intercept at the GP cooperative level. Additional analysis adjusting for compliance will also be performed (access to booklet vs. actual use). During data analysis, researchers will be blinded to the group assignments.

### Ethics and dissemination

All data will be obtained, managed and monitored according to the guidelines of good clinical practice. This study was approved by the ethics committee of Zuyderland-Zuyd (METC Z) in Heerlen, The Netherlands (reference 14-N-171) and is reported in accordance with the Standard Protocol Items: Recommendations for Interventional Trials (SPIRIT) guidelines [22]. The SPIRIT checklist is provided in Additional file 1, and the SPIRIT figure is graphically represented in Table 1. Findings of the study will be published and the results disseminated regardless of the magnitude or direction of effect.

## Discussion

The aim of this study is to optimise management of febrile children during GP out-of-hours care by giving GPs access to an illness-focused interactive booklet to be used during consultations for childhood fever and common infection in the out-of-hours setting. Illness in this light refers to the subjective response of the patient, or in this case the parents of the child who is unwell: how the parents perceive the origin and significance of this event, how it affects their behaviour, and the steps they take to remedy this situation [23]. Previous research has shown that alongside specific symptoms that often accompany fever, the decision to consult a GP during out-of-hours care is driven by parental needs for reassurance and reliable, consistent information on self-management strategies on one hand, and by non-medical factors such as work during the day and the fact that fever typically rises during the early evening on the other hand [9]. In turn, GPs acknowledge that this decision that parents make to consult during out-of-hours care plays an important role in the decision to prescribe antibiotics [11]. Exploring the illness experience of parents of children with fever and infections may lead to a potential intervention which can improve these consultations because it specifically addresses the concerns and questions that parents have when their child is sick. However, strong safety net advice provided in a booklet can hypothetically also provide a disease-focused solution for GPs by providing them with a way to reduce diagnostic uncertainty with these children, thereby also reducing the number of ‘better safe than sorry’ antibiotic prescriptions [15].

Providing parents of febrile children with safety net advice during consultations has been proposed previously [12, 24]. However, this will be the first study to explore the impact of using an illness-focused interactive booklet on antibiotic prescriptions, (re-)consultations and intention to re-consult, referral rates, parental satisfaction and self-reported adverse events in febrile children during out-of-hours GP care. An interactive booklet has been shown to be a promising intervention for reducing antibiotic prescriptions in different populations and settings in primary care [13, 14]. For example, one study of children with respiratory tract infections showed a 50% reduction in antibiotic prescriptions [14]. The clinically relevant difference of 6% chosen in this study is arbitrary because this is the first such study in this setting (out-of-hours care) and also in The Netherlands. Additionally, baseline prescription rates differ widely between different settings and countries, and baseline prescription rates in The Netherlands are already lower than in many other Western countries. As mentioned, the difference chosen is based on the fact that it will reduce the number of prescriptions to from one in four to one in five to six. However, we believe that any significant reduction can be considered clinically relevant in an era of increasing antibiotic resistance.

Previous research showed that GPs believe that in order to make an intervention suitable for use during out-of-hours care, it needs to be physically available in every consultation room [11] because this can act as a reminder to use the intervention, but specifically to avoid having to go through the effort of downloading or printing material in these often time-pressured consultations. This was an important reason to choose a physical booklet during this study. If the booklet turns out to be successful and satisfactory, it is our intention to spread it digitally as well as physically.

Every participating GP out-of-hours cooperative is randomised either to GP access to the illness-focused interactive booklet or to care as usual. In this case, we believe this design has multiple advantages over an individual RCT. It is inappropriate to randomise the intervention on an individual level because of the high risk of contamination. To clarify, communication skills cannot be randomised on a patient level, because it would be very demanding for GPs to change communication between every patient. Moreover, GPs can become confused when they have to use different communication skills with different patients. This would result in a risk of exposing parents in the control group to information from the intervention and creating the risk of contamination. This risk is especially high because GPs do not see febrile children at a fixed rate. To explain, we anticipate that GPs will be triggered by the content of the booklet to improve how they provide information within the consultation. If the trial were individually randomised, then there would be a risk that GPs would improve the information they provided to parents in the control group as well. We also believe it is not feasible to randomise on a GP level. A Dutch GP has approximately 12–40 shifts per year, and 50% of the consultations will not be eligible for recruitment, because no young children are physically seen. In other words, if a GP has only 12 shifts annually and only 50% of the consultations are eligible for recruitment, the chances of that GP actively remembering to hand a booklet to parents are small, especially if not every GP at one cooperative is working with the booklet. Besides this fact, it is also more practical to provide every consultation room with the necessary material, thereby making use of the tool more attractive, accessible and pragmatic in often time-pressured consultations.

However, using a cluster RCT also has its limitations. First, blinding of the participating GPs is very difficult because the transfer from care as usual to the intervention is obviously noticeable. To avoid bias, we will blind GPs to the outcome in both groups. Additionally, those with access to outcome data will be blinded by coding the dataset. Second, randomisation takes place on a GP out-of-hours cooperative level, and the cluster effect has to be taken into account. It is possible that participants within one cluster share certain characteristics, such as quality of care at the GP out-of-hours cooperative, which might result in a substantial loss of power. Therefore, we choose to correct for the cluster effect in the sample size calculation and in the data analysis by using multilevel analysis. Estimation of a required sample size in cluster RCTs is difficult because the expected effect size, anticipated cluster size and ICC have to be estimated and reported [25]. Despite these unknown variables, we believe this is the best methodology for this pragmatic study [26].

It can be expected, that out-of-hours cooperatives not receiving the illness-focused interactive booklet during the intervention may progressively lose interest in including patients. To prevent this, those centres allocated to the control arm will be informed that they will receive the information exchange tool after the study period as an incentive.

The average antibiotic prescription rate found in our cohort study, which is chosen as the baseline antibiotic prescribing proportion, is less than the 35% antibiotic prescription rate found in previous studies in adults and children [5, 14, 27]. As we know from previous studies, antibiotic prescription rates vary extensively between GPs. Therefore, we have purposefully chosen to use broad inclusion criteria and not to select specific causes of fever in children. By doing so, we aim to get as close as possible to actual practice and considerations of GPs’ prescribing decisions. This means that we expect that not every child in the intervention group will receive the booklet, owing to various realistic reasons such as a language barrier, a specific disease that is not described in the booklet, or because parents simply do not wish to receive the booklet. Moreover, GPs will differ in their own perceived need to use such an interactive booklet during consultations. While some may use it in all their consultations for children with fever, some may never use it. This probably reflects the use of current information materials, mostly patient leaflets [13]. We choose to perform a pragmatic study, allowing for this variation but also facilitating possible future implementation in daily practice.

### Trial status

The trial is registered with ClinicalTrials.gov (NCT02594553), and recruitment was ongoing during the time of first submission.

## Additional file

Additional file 1: SPIRIT checklist. (DOC 120 kb)

## Abbreviations

CHILI: Childhood Infections Limburg study
GP: General practitioner
ICC: Intra-cluster correlation coefficient
RCT: Randomised controlled trial
SPIRIT: Standard Protocol Items: Recommendations for Interventional Trials
VAS: Visual analogue scale
